# Chronic DeBakey Type IIIB Aortic Dissection Presenting Acutely: A Diagnostic and Surgical Challenge in an Elderly Hypertensive Patient

**DOI:** 10.7759/cureus.85310

**Published:** 2025-06-03

**Authors:** José Manuel García Romero, Daniela De Noriega Guzmán, Francisco Ortega Arreola, Elizabeth Alcala, Omar Reyes

**Affiliations:** 1 General Practice, Autonomous University of Querétaro, Querétaro, MEX; 2 Surgery, Hospital Zambrano Hellion TecSalud, San Pedro Garza García, MEX; 3 Surgery, General Hospital of Querétaro, Querétaro, MEX; 4 Surgery, XXI Century National Medical Center, Mexican Social Security Institute (IMSS), Mexico City, MEX; 5 Surgery, General Hospital Zone 1-A, Mexican Social Security Institute (IMSS), Mexico City, MEX

**Keywords:** aortic aneurysm, aortic dissection, debakey type iiib, emergency surgery, hypertension, pseudoaneurysm, thoracoabdominal aneurysm, type b dissection

## Abstract

Aortic dissection is a rare but life-threatening condition requiring prompt diagnosis and intervention. We report the case of a 74-year-old woman with a history of long-standing hypertension who presented with sudden-onset tearing abdominal and lower back pain. Initial evaluation revealed hypotension, diminished femoral pulses, and a diastolic murmur, raising suspicion for acute aortic pathology. Imaging confirmed a chronic DeBakey type IIIB (Stanford type B) aortic dissection extending from the left subclavian artery to the iliac arteries, associated with aneurysmal dilation, a saccular pseudoaneurysm, extensive atheromatosis, and signs of malperfusion. Emergent surgical repair with a multi-branched aortic graft and valve-sparing aortic root replacement was successfully performed. This case highlights the diagnostic challenges posed by chronic dissections presenting acutely, the limitations of relying solely on aortic diameter for risk assessment, and the critical importance of early recognition and comprehensive imaging in guiding timely, life-saving interventions.

## Introduction

Aortic dissection is a life-threatening condition classified under the umbrella of acute aortic syndromes (AAS). It results from a tear in the intimal layer of the aorta or from bleeding within the aortic wall due to rupture of the vasa vasorum, leading to separation of the aortic wall layers [1].

Recent epidemiological data estimate the incidence of acute aortic dissection at three to five cases per 100,000 individuals per year. However, this figure is likely underestimated, as most studies do not account for pre-admission deaths [2]. Despite its rarity, aortic dissection is a catastrophic event, with mortality rates reaching up to 90% within the first 40 days after symptom onset [3].

Several risk factors have been associated with aortic dissection. These include connective tissue disorders (such as Marfan syndrome, Loeys-Dietz syndrome, and type IV Ehlers-Danlos syndrome), male sex (with a male-to-female ratio of 2-4:1), and age (approximately 75% of cases occur in individuals aged 40 to 70 years) [4,5]. Cardiovascular risk factors, such as dyslipidemia, hypertension, elevated apolipoprotein A levels, and smoking, are also significant contributors [2]. Additional predisposing conditions include aortitis (from infectious or non-infectious causes like Takayasu arteritis and giant cell arteritis), trauma, pregnancy, bicuspid aortic valve, aortic coarctation, aortic root dilation, and the presence of aortic aneurysm [6]. The risk of dissection increases proportionally with aortic diameter, with complications occurring in up to 30% of patients once the diameter reaches 60 mm. Although aortic dilation heightens the risk, it is not a prerequisite for dissection [7].

Aortic dissection is primarily classified based on anatomical location and symptom onset, with the two most widely used classification systems being Stanford and DeBakey. In the Stanford system, Type A dissections involve the ascending aorta, while Type B dissections do not. The DeBakey system further subdivides dissections as follows: Type I involves both the ascending and descending aorta, Type II is limited to the ascending aorta, and Type III is confined to the descending aorta [8]. 

The clinical presentation varies depending on the extent and location of the dissection, with symptoms corresponding to the structures involved. Acute type B aortic dissection typically presents with sudden, severe, sharp, or tearing chest or back pain. Due to its variable presentation, it can be easily misdiagnosed among patients with acute chest pain, necessitating a high index of suspicion. Imaging is essential for confirming the diagnosis, identifying an intimal tear, determining the anatomical classification, and assessing for valvular or branch involvement. Current guidelines recommend either CT aortography or transesophageal echocardiography (TEE) for definitive diagnosis [2,4,5].

This report presents a rare case of Stanford Type B/DeBakey Type III aortic dissection, associated with aortic aneurysms and diffuse atheromatosis. It aims to raise awareness among emergency physicians to consider this diagnosis early in patients presenting with acute chest or back pain, facilitating prompt recognition and treatment.

## Case presentation

A 74-year-old female with a known history of long-standing systemic arterial hypertension, managed with losartan 50 mg twice daily and metoprolol 50 mg once daily, presented to the emergency department with acute onset of severe abdominal and lower back pain. The pain was described as sudden, tearing in nature, and radiating from the epigastrium to the lumbar region. She reported progressive dyspnea on exertion over the past several months and had been experiencing occasional episodes of chest discomfort, orthopnea, and lower extremity edema, which she had attributed to aging.

On arrival, the patient was in acute distress. Vital signs revealed hypotension with a blood pressure of 80/50 mmHg, tachycardia at 120 bpm, respiratory rate of 24 breaths per minute, and oxygen saturation of 92% on room air. Physical examination showed diaphoresis, cool extremities, and diffuse abdominal tenderness with guarding. Femoral pulses were diminished bilaterally, raising concern for aortic pathology. Cardiac auscultation revealed a diastolic murmur at the right upper sternal border and a holosystolic murmur best heard over the lower left sternal border.

Initial laboratory workup demonstrated normocytic anemia, elevated lactate levels, and mild leukocytosis. Electrocardiogram showed sinus tachycardia without ischemic changes. A bedside transthoracic echocardiogram (TTE) revealed significant aortic root dilation and severe aortic regurgitation, as well as moderate tricuspid regurgitation. Biventricular systolic function was preserved with an estimated left ventricular ejection fraction of 65%. There was evidence of concentric left ventricular hypertrophy, consistent with hypertensive heart disease. The right ventricular systolic pressure was estimated at 33 mmHg, suggestive of moderate pulmonary hypertension.

**Figure 1:**
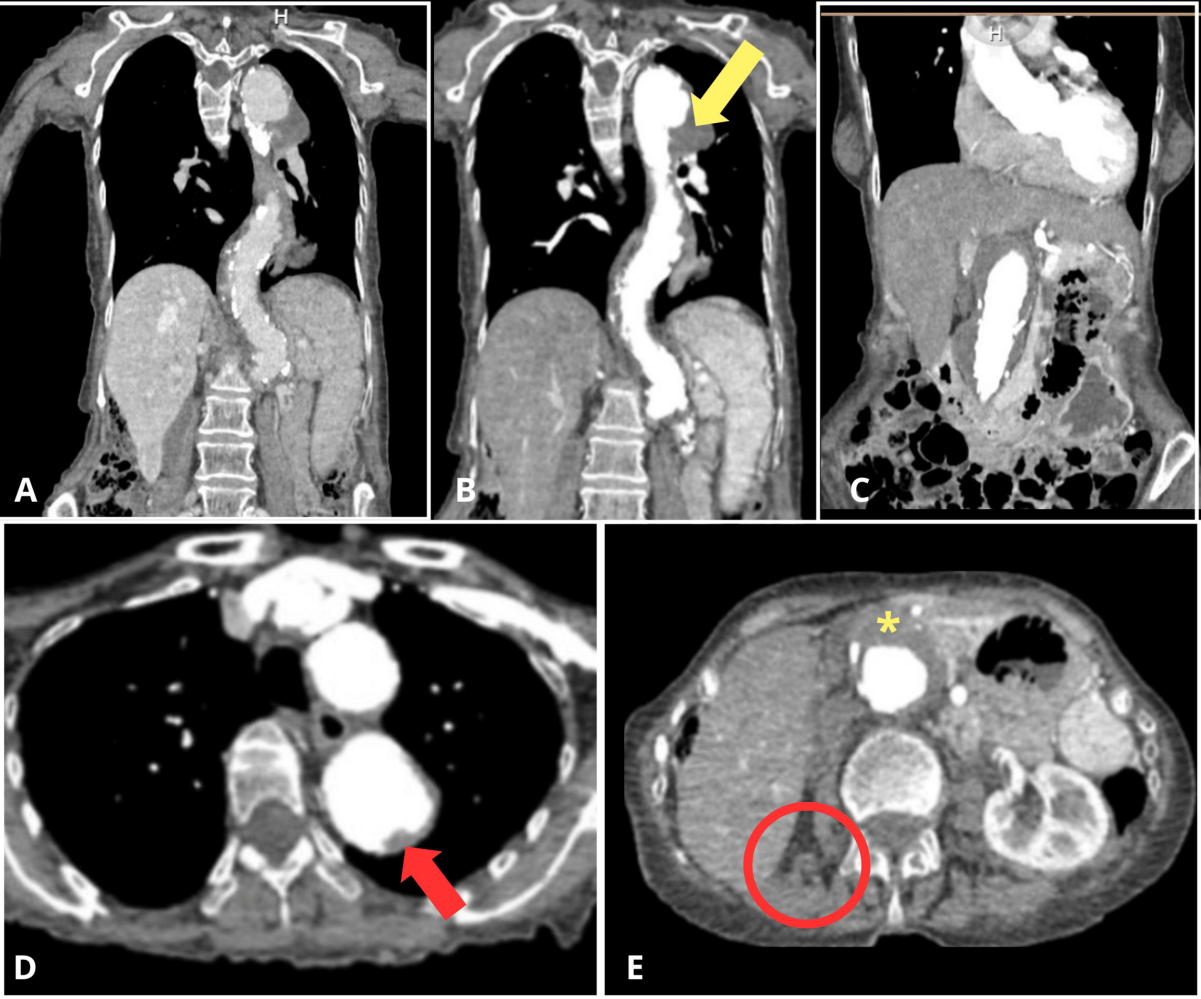
Contrast-enhanced computed tomography A: Coronal CT angiography demonstrates a DeBakey type IIIB aortic dissection, with fusiform dilation of the descending thoracic and abdominal aorta as well as multiple calcifications. B: Coronal contrast-enhanced CT angiography, a hypodense lesion consistent with saccular aneurysmal degeneration is identified at the level of the descending thoracic aorta. The aneurysmal sac contains an intramural thrombus, as evidenced by the heterogeneous density within the aneurysm (yellow arrow). C: Coronal contrast-enhanced CT shows an intramural thrombus in the distal abdominal aorta, extending caudally to near the aortic bifurcation. It appears as a well-defined hypodense area along the posterior wall, without significant luminal narrowing, dissection, or contrast extravasation. D: Axial CT angiography reveals an intramural thrombus (indicated by the red arrow), originating just distal to the origin of the supra-aortic branches. E: Axial CT angiography reveals right renal atrophy (red circle), with no contrast enhancement of the right renal artery or renal parenchyma. An intramural thrombus in the abdominal aorta is indicated by a yellow asterisk.

Given the high clinical suspicion of aortic dissection or rupture, an urgent contrast-enhanced CT angiography of the chest, abdomen, and pelvis was performed. The scan revealed a chronic DeBakey type IIIB aortic dissection. The dissection extended from the origin of the left subclavian artery to the iliac arteries and was associated with aneurysmal dilation of the ascending aorta (4.7 cm), as well as the descending thoracic and abdominal aorta (up to 4.6 cm). A 3 cm saccular pseudoaneurysm with internal thrombus was noted in the upper descending thoracic aorta. Extensive atheromatous calcifications and intraluminal thrombus contributed to segmental stenoses (30-40%) of the abdominal aorta. Additional findings included tracheal deviation due to aortic compression, cardiomegaly with coronary and valvular calcifications, and an atrophic right kidney with simple cysts and lithiasis. No signs of active rupture were identified (Figure 1). 

Despite the absence of radiological signs of rupture or active bleeding, urgent surgical management was required due to the extensive aortic involvement and multiple areas of calcification and intramural thrombus.

The patient was immediately transferred to the intensive care unit for hemodynamic stabilization with intravenous fluids and blood products. Cardiothoracic and vascular surgery teams were consulted, and the patient underwent emergent surgical intervention. During surgery, an open approach via left thoracotomy was performed, exposing the thoracoabdominal segment of the descending aorta. Proximal and distal aortic clamping was carried out to achieve vascular control, followed by opening of the aneurysmal sac and resection of the affected segment. Due to extensive involvement of major visceral branches, reconstruction of the superior mesenteric artery and left renal artery was performed using end-to-side anastomoses with a multi-branched Dacron prosthetic graft. The right renal artery was not reconstructed due to advanced renal atrophy.

Intraoperative findings confirmed the presence of a thoracoabdominal aneurysm with extensive involvement of the visceral segment. A complex aortic graft repair was performed using the multi-branched prosthetic graft. The procedure included reconstruction of the visceral arteries and replacement of the involved aortic segments. Aortic valve inspection revealed functional compromise due to annular dilation, contributing to the severe regurgitation observed on imaging. A valve-sparing aortic root replacement was performed concurrently.

The patient was monitored postoperatively in the intensive care unit with mechanical ventilation, vasopressor support, and close neurovascular surveillance. Over the following days, she demonstrated gradual clinical improvement, with stabilization of vital signs and recovery of renal function. 

## Discussion

Thoracoabdominal aortic aneurysms (TAAAs) involving chronic type IIIB dissection, as presented in our patient, represent a particularly complex subset of aortic pathologies. Although classified as chronic in this case, the clinical presentation mimicked an acute process, emphasizing the challenges in recognizing and managing these entities, especially in elderly patients with multiple comorbidities.

The clinical presentation of our patient shared notable features with those described in Pérez-Camargo et al.’s review of AAS. Our patient presented with classic symptoms: abrupt onset of severe, tearing abdominal and back pain radiating to the lumbar region, hypotension, and peripheral pulse deficits. According to Pérez-Camargo et al., chest or dorsal pain is the most common presenting symptom in AAS, reported in up to 94% of cases, with a characteristically sudden onset and severe intensity, which closely mirrors the clinical profile in our case [3]. The presence of diminished femoral pulses and hypotension further supports aortic dissection as a high-priority differential diagnosis, aligning with high-specificity signs (91-95%) noted in the literature [2,3]. 

Imaging findings played a pivotal role in both the diagnosis and classification of our patient’s condition. Pérez-Camargo et al. emphasized the utility of contrast-enhanced CT as the gold standard for AAS, with sensitivity and specificity exceeding 95% and 98%, respectively [3,7]. CT allowed for detailed assessment of the dissection’s extent, the identification of the pseudoaneurysm, and the degree of aneurysmal degeneration in our patient, elements critical for surgical planning and risk stratification. Notably, while no active rupture was observed, the presence of a saccular pseudoaneurysm with internal thrombus suggested high-risk instability, warranting emergent intervention despite the absence of extravasation.

Classification systems provide essential guidance for both the diagnosis and treatment of aortic dissections. In our case, the dissection was categorized as DeBakey type IIIB, involving the descending thoracic and abdominal aorta beyond the left subclavian artery, without involvement of the ascending aorta. According to Gawinecka et al., the DeBakey and Stanford systems remain the standard for anatomical classification, with type IIIB corresponding to Stanford type B dissections [2,3,8]. However, more complex systems, such as the TEM classification (Type, Entry site, Malperfusion), have been developed to incorporate both anatomical features and perfusion-related criteria [3]. If applied to our case, the dissection would be classified as Type B, with an entry point likely in the descending thoracic segment (E3), and M3 malperfusion, given the involvement of visceral arteries and renal ischemia-evidenced by the atrophic kidney and systemic hypoperfusion.

An important consideration in our patient's case is the diagnostic and prognostic value of aortic diameter, particularly in the setting of aortic dissection. Traditional surgical thresholds have focused on an ascending aortic diameter ≥5.5 cm as the primary indicator for elective intervention in non-Marfan patients. However, the findings of Pape et al. challenge this paradigm, demonstrating that nearly 60% of patients with acute type A dissection presented with an aortic diameter below this threshold, and 40% had diameters under 5.0 cm. These data suggest that relying solely on diameter as a surgical trigger may fail to prevent a significant portion of dissections [7].

Our patient’s ascending aortic diameter was 4.7 cm, which would not have met current guideline thresholds for elective surgery. Yet, she presented with a complex chronic DeBakey type IIIB dissection, aneurysmal degeneration, and severe aortic regurgitation, leading to hemodynamic instability. This case exemplifies how aortic size alone is insufficient to capture the true rupture or dissection risk, especially when compounded by chronic systemic hypertension, valvular dysfunction, and signs of pseudoaneurysm instability.

The International Registry of Acute Aortic Dissection (IRAD) study further emphasizes that hypertension, older age, and radiating pain were independent predictors of dissection at smaller diameters, all of which were present in our patient. Interestingly, the study also noted that mortality was not significantly associated with aortic diameter, reinforcing the need for improved risk stratification tools beyond simple anatomical measurements [4,7].

Interestingly, Pérez-Camargo et al. emphasize that chronic dissections, while classically defined as occurring more than 90 days after symptom onset, can still present acutely if complicated by pseudoaneurysm expansion, malperfusion, or valvular involvement. Furthermore, the absence of contrast extravasation on CT does not exclude life-threatening instability, particularly when pseudoaneurysm or valvular dysfunction is present. Prompt recognition and surgical intervention remain paramount, as highlighted by both our case and the current literature [3]. This is consistent with our patient, who had a chronic dissection but experienced acute hemodynamic deterioration, likely precipitated by pseudoaneurysm instability and severe aortic regurgitation secondary to annular dilation.

The surgical repair of thoracic aortic aneurysms and dissections is highly individualized, depending on anatomical involvement, associated anomalies, and patient stability. Both our case and those reported by Narita et al. involved complex thoracic aortic pathology, yet the surgical approaches differed significantly due to anatomical considerations, urgency, and patient-specific factors.

In our patient, a chronic DeBakey type IIIB dissection with aneurysmal degeneration extending into the abdominal aorta, complicated by a saccular pseudoaneurysm and severe aortic regurgitation, necessitated emergent surgical intervention. A left thoracoabdominal approach enabled resection of the diseased aortic segment and reconstruction with a multi-branched prosthetic Dacron graft. Visceral artery involvement required selective revascularization, and a valve-sparing root replacement was performed to address annular dilation and severe regurgitation. This comprehensive single-stage procedure aligns with international recommendations for open repair in cases of complicated DeBakey IIIB dissections with visceral malperfusion and pseudoaneurysm formation [1,9].

In contrast, the patients described by Narita et al. underwent elective surgical repairs, which allowed for meticulous preoperative planning using 3D-reconstructed CT imaging, particularly necessary in the presence of an aberrant right subclavian artery (ARSA). In Case 2, which most closely resembles our case regarding dissection type, the patient also had a chronic DeBakey IIIB dissection and underwent total descending thoracic aortic replacement via left thoracotomy [9]. The surgical focus in Narita’s case was on preserving the ARSA and reconstructing the left subclavian artery, with the use of deep hypothermic circulatory arrest (DHCA) and retrograde cerebral perfusion (RCP) employing the Takamoto method [9]. Unlike our patient, however, there was no visceral artery involvement or need for concurrent aortic root intervention, underscoring the increased complexity of our case.

Another key differentiator is the urgency of intervention. Narita’s cases were managed electively, guided by progressive aneurysmal dilation and stable clinical status, enabling individualized graft planning and operative timing. In contrast, our patient presented with acutely worsening symptoms suggestive of impending rupture, prompting immediate surgical intervention despite the absence of contrast extravasation. The extent of visceral involvement in our case required a broader operative field and multi-visceral reconstruction, not observed in the Narita series.

The long-term management of chronic DeBakey type IIIB dissecting aortic aneurysms remains challenging, with surgical approach significantly impacting outcomes. Our patient’s emergent thoracoabdominal aortic aneurysm repair (TAAAR), combined with a valve-sparing root procedure, highlights the feasibility and potential benefit of an aggressive open strategy in select cases. This approach is supported by findings from Miura et al., who demonstrated that TAAAR provides superior long-term durability. Their study reported a 10-year aortic event-free survival of 93.8% for TAAAR, compared to only 51.4% at seven years with thoracic endovascular aortic repair (TEVAR), which also had a markedly higher reintervention rate (45.5%) [10].

Although open TAAAR carries greater perioperative risk, including prolonged ventilation and higher transfusion requirements, our case supports its utility in the setting of complicated chronic dissections with visceral malperfusion and aortic valve dysfunction [10]. The favorable early recovery observed in our patient underscores the importance of individualized, anatomy-driven decision-making in managing these high-risk cases.

Although open TAAAR carries greater perioperative risk, including prolonged ventilation and higher transfusion requirements, our case supports its utility in the setting of complicated chronic dissections with visceral malperfusion and aortic valve dysfunction [10]. The favorable early recovery observed in our patient underscores the importance of individualized, anatomy-driven decision-making in managing these high-risk cases. While TEVAR offers benefits in terms of lower invasiveness and reduced initial morbidity, its medium- and long-term outcomes are inferior, particularly in chronic dissections with a perfused false lumen. Therefore, TAAAR or descending thoracic aortic replacement (DTAR) should be considered as primary approaches, tailored to patient-specific factors, rather than adopting TEVAR as a first-line treatment for all cases of chronic DeBakey type IIIB dissecting aneurysms. Endovascular repair may be considered in select cases where open surgery is contraindicated due to high operative risk, but its limitations must be clearly acknowledged [10].

## Conclusions

This case illustrates the complexity of managing thoracoabdominal aortic aneurysms in the context of coexisting severe valvular disease. Prompt diagnosis, coordinated surgical intervention, and individualized perioperative care were essential to ensuring the patient’s survival and recovery. Future management will focus on addressing the remaining valvular pathology in a controlled, elective setting.

Additionally, the case underscores the diagnostic challenges and therapeutic urgency associated with chronic DeBakey type IIIB dissecting aortic aneurysms, particularly when acute symptoms arise in the absence of overt rupture. Although our patient did not meet standard imaging criteria for rupture, the presence of high-risk features, including pseudoaneurysm formation, visceral involvement, and severe aortic regurgitation, warranted emergent surgical intervention. Her successful outcome following thoracoabdominal aortic aneurysm repair with valve-sparing aortic root replacement highlights the importance of tailoring surgical decisions to clinical instability and anatomical complexity rather than relying solely on imaging findings.
